# Review of Evidence for the Usage of Antioxidants for Eye Aging

**DOI:** 10.1155/2022/5810373

**Published:** 2022-10-03

**Authors:** Priscilla Peixi Choo, Pui Juan Woi, Mae-Lynn Catherine Bastion, Rokiah Omar, Mushawiahti Mustapha, Norshamsiah Md Din

**Affiliations:** ^1^Department of Ophthalmology, Faculty of Medicine, Pusat Perubatan UKM, Jalan Yaacob Latif, 56000 Cheras, Kuala Lumpur, Malaysia; ^2^Program of Optometry and Vision Science, Faculty of Health Sciences, Universiti Kebangsaan Malaysia, Jalan Raja Muda Abdul Aziz, 50300 Kuala Lumpur, Malaysia

## Abstract

Oxidative stress is one of the common factors leading to age-related eye diseases in older adults. Factors such as high oxygen consumption, high concentration of polyunsaturated fatty acids, and cumulative exposure to high-energy visible light in the eyes, lead to excessive generation of reactive oxygen species, hence triggering apoptosis of ocular cells and giving rise to ophthalmic diseases. Dietary supplements such as carotenoids, anthocyanins, and vitamins have antioxidant properties which may be of benefit in retaining better vision or reversing vision impairment; thus, studies have been conducted to understand the role of dietary supplements in the treatment or prevention of ophthalmic diseases. While high concentration of carotenoids such as lutein and zeaxanthin decrease the risk of developing age-related macular disease, anthocyanins and vitamins play a role in the treatment and prevention of other ophthalmic diseases: saffron extract reduced intraocular pressure in glaucoma patients; bilberry extract prevented impairments in lenses and retina, as well as alleviate symptoms of dry eye disease; high concentration of beta-carotene may reduce the risk of developing cataract. Further studies with clinical measurements are required to investigate the effectiveness of antioxidants on visual function and ophthalmic diseases.

## 1. Main Text

Proper eye healthcare is a vital component of maintaining one's overall health. Negligent care of the eyes contributes to a multitude of ophthalmic diseases, resulting in impaired vision and affecting daily activities. These diseases may be minor in themselves, resulting in relatively insignificant symptoms such as slight inflammation or irritation. However, over time, more serious diseases may result in significant eye damage, decreased quality of vision, or total blindness.

Approximately 250 million people worldwide suffer from varying degrees of vision loss [1]. The leading causes of vision loss are common eye conditions such as cataracts, age-related macular degeneration (AMD), glaucoma, and diabetic retinopathy (DR), which largely affect older adults. While the origins and causes of age-related eye diseases are complex and multifactorial, oxidative stress has been implicated as a common conducive mechanism. The eye is susceptible to oxidative stress due to its high oxygen consumption, high concentrations of polyunsaturated fatty acids, and cumulative exposure to high-energy visible light. These factors combined lead to excessive generation of reactive oxygen species (ROS), which are free radicals containing oxygen, and can trigger oxidative damage to deoxyribonucleic acid (DNA), proteins, and lipids [2], inducing apoptosis of ocular cells and resulting in ophthalmic diseases [3]. It has been hypothesized that antioxidants may be of benefit in retaining better vision or even reversing vision impairment. Therefore, there is significant research interest in the role of dietary antioxidants and the potential therapeutic benefits of antioxidant supplements as a simple and cost-effect strategy for disease prevention and/or control [4–7]. This review will focus on the antioxidant properties of carotenoids (lutein and zeaxanthin), anthocyanins (saffron and bilberry), and vitamins (vitamin A) in the context of treating or preventing age-related eye diseases.

### 1.1. Evidence for Carotenoids

Xanthophyll (oxygen-containing) carotenoids, including lutein and zeaxanthin, are selectively concentrated in vision-related tissues (the eye and brain) [8]. Nearly all human ocular structures except the vitreous, cornea, and sclera contain lutein, zeaxanthin, and metabolites. The macula contains lutein and zeaxanthin with concentrations up to 100-fold higher than elsewhere in the eye [9]. Macular carotenoids are estimated to absorb 40 to 90% of incident blue light (depending on concentration) [10], protecting the retina from light-related damage [11] and reducing light scatter. The presence of oxidized metabolites in the retinal pigment epithelium (RPE), lens, ciliary body, and iris also suggests that lutein and zeaxanthin are protective against oxidative stress [12]. Lutein and zeaxanthin have also been found to prevent the increase in oxidation-induced cytokines and upregulate the expression of inflammation-related genes [13, 14]. In addition to its retinal effects, intake of a lutein supplement has been found to lower circulating levels of a rate-limiting enzyme in the alternative complement activation pathway, which itself plays an important role in the inflammatory response and the development of AMD [15, 16].

Several studies have demonstrated evidence of an inverse relationship between the presence of lutein and zeaxanthin in the retina and the risk for AMD [17–21]. An increase in macular pigment optical density (MPOD) level, where approximately 16.8 to 27.9% change in MPOD (*P* < 0.001), and a small nonsignificant improvement in visual acuity (VA) have been found in AMD patients who received oral lutein supplements for six months [22] and over one year [23]. An electroretinographic study has shown that the use of carotenoid supplements containing lutein, zeaxanthin, and meso-zeaxanthin may improve the visual function of type 2 diabetes patients [24], where best-corrected visual acuity (BCVA) remained normal (≥9/10) in each eye, while mean central foveal thickness (CFT) increased approximately 5 to 7 *μ*m in each eye (*P* < 0.001). Multifocal electroretinography (mfERG) has revealed a significantly increased retinal response density surrounding the fovea (all central 3 rings) in both eyes two years after carotenoids supplement intake in type 2 diabetes patients (*P* < 0.001). Additionally, daily intake of lutein supplements in healthy individuals also showed increased MPOD levels alongside improvement in contrast and glare sensitivity [25]; with a significant increase in MPOD levels at the more central measured eccentricities after 6 months of intervention, significant improvement in contrast sensitivity after 3 months of intervention, and change in glare sensitivity threshold after a year of intervention.

Higher lutein and zeaxanthin levels in the diet or blood are associated with decreased risk of macular disease [26–35]. However, many factors affect the degeneration of macular pigments, including physical inactivity, poor diet, low levels of lutein and zeaxanthin in the diet and serum, smoking, components of metabolic syndrome (i.e., obesity, diabetes, and hypertension), and common variants in genes related to macular pigment optical density (MPOD) and/or serum lutein and zeaxanthin [36]. These factors help in predicting the odds of developing early/intermediate AMD [37].

A reduction in genetic risk for AMD among individuals with high lutein and zeaxanthin intake has been observed among several cohorts (the Rotterdam Study [38], a pooled analysis of the Rotterdam and Blue Mountain Eye studies [39], and Carotenoids in Age-related Eye Disease Study (CAREDS) [40]). Despite the lowest rates of AMD being reported in individuals consuming 5 to 6 mg/day [41–44], diets high in lutein and zeaxanthin are also high in other carotenoids which may contribute to lower risk [44]. Lutein and zeaxanthin supplements alone did not influence AMD progression or have significant changes in BVCA over three years [45], as a significant increase in macular pigment (MP) was observed despite having significant improvements in contrast sensitivity at 36 months of intervention. However, slowed AMD progression was seen when combined with other antioxidant supplements [46]. Average BCVA in patients receiving an oral preparation containing lutein, zeaxanthin, vitamin C, vitamin E, copper, and zinc was approximately 4.8 letters better than the placebo group after 36 months (*P* < 0.05). While these patients showed insignificant improvement in contrast sensitivity, slower AMD progression along a morphologic severity scale was observed.

The results of primary analyses in the Age-Related Eye Disease Study 2 (AREDS2) [47] trial suggest that including 10 mg of lutein and 2 mg of zeaxanthin in high-dose antioxidant supplements does not lower the progression to advanced AMD, as the hazard ratio (HR) compared with placebo were 0.89 to 0.97 (*P* > 0.05) in all intervention groups. However, secondary analyses [48] have indicated a modestly lower five-year risk of progression in participants who began the study with low levels of lutein and zeaxanthin in their diets, or when the antioxidant supplements did not contain beta-carotene, as the hazard ratio appear to be less than 1 during the exploratory analyses and in eyes with bilateral large drusen at baseline (*P* < 0.05 for development of late AMD and neovascular AMD). While there is no recommended daily intake for lutein and zeaxanthin, the intake of lutein is recommended to be higher than zeaxanthin among all age groups [49]. The health benefits of a daily intake of 10 mg of lutein and 2 mg of zeaxanthin have been shown in a randomized, double-blind, placebo-controlled human study [28].

Exposure to light at the retinal level increases phagocytosis in the outer segment (OS) of photoreceptors and induces the formation of ROS by RPE cells [50], followed by photooxidative stress in the retina when an imbalance develops between ROS and endogenous antioxidative systems [51]. Moreover, the impaired retinal antioxidant status may lead to the overexpression of proinflammatory and angiogenic mechanisms, which accounts for injured retinal microvasculature [52]. Nutritional supplements such as polyphenol and xanthophyll that scavenge ROS have been shown to prevent or delay the progression of early AMD by direct interaction with rhodopsin, modulate visual pigment function, and protect retinal cells from oxidative stress-induced cell death [53, 54]. Anthocyanins have been found to act directly as antioxidants to neutralize ROS by donating hydrogen ions and modulating cell signaling pathways [55], as well as effects on anti-inflammatory and antiapoptotic pathways and gene expression [56].

### 1.2. Evidence for Saffron

*Crocus sativus* L. (Iridaceae), commonly known as saffron, has been studied for its main components (crocins, crocetin, picrocrocin, and safranal) and its potent antioxidant activity [57–60], as well as the binding capacity of saffron metabolites to biomolecules, which protects them from free radicals [57, 61]. Saffron components have also been found to possess anti-inflammatory and antiapoptotic effects, possibly by the inhibition of caspase-mediated apoptosis after retinal damage [62–65], as well as to increase oxygen diffusion and improve retinal and choroidal blood flow [66, 67]. Studies on saffron extract suggest that it may exert a protective effect in patients with glaucoma. Oral consumption of aqueous saffron extract for three weeks was able to reduce intraocular pressure (IOP) in primary open-angle glaucoma [68], from a mean IOP of 12.9 ± 3.7 mmHg to 10.9 ± 3.3 mmHg after three weeks of intervention as compared to the control group (mean baseline IOP of 14.0 ± 2.5 mmHg and 13.5 ± 2.3 mmHg after three weeks). Hydrophilic saffron extracts standardized to 3% crocin was also found to decrease the neuroinflammation associated with increased IOP by decreasing microglion numbers and morphological signs of their activation including soma size and process retraction, as well as reversed ocular hypertension induced down-regulation of P2RY12, preventing retinal ganglion cell death in a glaucoma model [69].

An electroretinographic study has shown an increase in the amplitude of “a” and “b” waves in rabbits with experimental retinal dystrophy on the fifth day after receiving an injection of the saffron extract [70]. Several studies have evaluated the impact of oral saffron supplementation (daily dose between 20 and 50 mg) on vision-related parameters in AMD patients. Both short-term and longer-term follow-ups demonstrated that saffron supplementation improved visual functions, despite variable formulation, doses, intervention durations, test methods, and outcome measures in these studies [60, 71–76]. Toxicology research has found that saffron is safe for human consumption. The dose of 30 mg/day seems efficacious, and toxic effects have been reported with 5 g or more, with a lethal dose of approximately 20 g [57, 77], although long-term and large-scale research works are yet to be conducted to confirm its effect on human health.

### 1.3. Evidence for Bilberry

Bilberry (*Vaccinium myrtillus* L.) from Ericaceae is particularly rich in anthocyanins, such as delphinidin, malvidin, petunidin, cyanidin, and peonidin [78], and it has been shown that bilberry anthocyanins modulate oxidative stress defense enzyme heme oxygenase-1 (HO-1) and glutathione S-transferase-pi in human RPE cells [79]. Fursova et al. found that long-term (1.5 to 3 months) supplementation with bilberry extract to OXYS rats affected by accelerated aging, which is associated with high sensitivity to oxidative stress, was effective in preventing degeneration. Approximately 70% of the control OXYS rats were found to have macular degeneration after 3 months of treatment, while bilberry supplementation prevented impairments in the lenses and retina [80]. Furthermore, Osada et al. found that bilberry extract attenuates photo-induced apoptosis and visual dysfunction, which is most likely due to ROS reduction and subsequent endoplasmic reticulum stress attenuation in a murine model of the photo-stressed retina (750 mg/kg body weight) [81].

Oral consumption of bilberry extract supplement for 8 weeks has been shown to reduce the video display terminals (VDT) load-induced ocular fatigue sensation including ocular pain, eye heaviness, uncomfortable sensation, and foreign body sensation, as reduced critical flicker fusion (CFF) was alleviated after 8 weeks of intervention (95% confidence interval, 0.10-1.60; *P* = 0.023), although no change in near point variation (NPA) was seen [82]. A longer period of consumption of bilberry extract (12 weeks) relieved the tonic accommodation of the ciliary muscle caused by VDT tasks and near-vision tasks, as the postload high-frequency component- (HFC-) 1 value at week 12 was significantly improved in the bilberry group (*P* = 0.017), with significantly better task load at week 12 (*Δ*HFC-1) at week 12 (*P* = 0.049) [83]. Bilberry extract may also alleviate the symptoms of dry eye disease (DED). The volume of tear secretion has shown improvement with bilberry extract supplement among subjects with DED over four weeks (*P* = 0.019), as well as a significant improvement of biological antioxidant potential (BAP, *P* = 0.003) and increased in modified BAP/diacron-reactive oxygen metabolites (d-ROMs) ratio, an indicator of overall balance between antioxidant potential and oxidative stress [84]. Apart from that, long-term consumption of bilberry extract can slow down axial elongation and control myopia progression in children with high myopia. Mean increase in spherical diopter was significantly lower (−10.8 ± 2.6 D to −11.2 ± 2.5 D) compared to controls (−10.5 ± 2.6 D to −12.3 ± 2.6 D). The increase in axial length was also significantly lesser (23.7 ± 1.2 mm to 23.9 ± 1.2 mm) compared to the control (23.9 ± 1.4 mm to 24.8 ± 1.5 mm) after 24 months [85]. A larger prospective study over a longer duration could reveal the true efficacy of these supplements against their cost.

### 1.4. Evidence for Beta-Carotene

Beta-carotene is a naturally occurring retinol (vitamin A) precursor obtained from certain fruits and vegetables with potential antineoplastic and chemopreventive activities. As an antioxidant, beta-carotene inhibits free radical damage to DNA and is typically associated with eye health [86]. The Age-Related Eye Disease Study (AREDS) demonstrated that daily oral supplementation with antioxidative vitamins and minerals (vitamin C, vitamin E, beta-carotene, zinc, and copper) reduced the risk of developing advanced AMD by 25% at five years (odds ratio (OR), 0.72; 99% confidence interval (Cl), 0.52-0.98), as well as a significant reduction in rates of at least moderate visual acuity loss (OR, 0.73; 99% Cl, 0.54-0.99) [86]. However, a secondary analysis of the Age-Related Eye Disease Study 2 (AREDS2) shows that more lung cancers are diagnosed in participants with beta-carotene intake than those without [47]. Despite epidemiologic studies showing no association between dietary intake of vitamin A and reduced risk of AMD [43, 87], high levels of vitamin A, *α*-carotene, lycopene, and lutein intake were associated with low risk of cataract. Risk of nuclear cataract was lowest in people with high plasma concentration of *α*-carotene (OR, 0.5; 95% Cl, 0.3-0.9, *P* = 0.006) or *β*-carotene (OR, 0.7; 95% Cl, 0.4-1.4, *P* = 0.033), risk of cortical cataract was lowest in people with high plasma concentration of lycopene (OR, 0.4; 95% Cl, 0.2-0.8; *P* = 0.003), and risk of posterior subcapsular cataract was lowest in people with high plasma concentration of lutein (OR, 0.5; 95% Cl, 0.2-1.0, *P* = 0.012) [88].

## 2. Recommended Clinical Measurements of Antioxidant Effects on Vision

Given the fact that most antioxidants are not considered essential nutrients, most health authorities have yet to establish recommended daily intakes. Factors such as diet, gender, the existence of food intolerances, and the quantities of nutrients in foods also affect the daily average intakes of individuals. While it is more beneficial to consume whole food sources as they contain various other nutrients, supplements are available in the market for individuals who require greater quantities of specific nutrients that they might otherwise lack or insufficiently consume. Lutein supplements were available ranging from 5 mg to 25 mg, some with <5 mg of zeaxanthin, while recommended dose for eye health is 10 mg of lutein per day and 2 mg of zeaxanthin per day [28]. Daily intake of 50 mg of anthocyanins per day was recommended by the Chinese authorities to reduce oxidative stress levels and consequently the risk of degenerative diseases [89], where 50–88.5 mg of saffron extracts and 80–100 mg of bilberry extracts is available in each capsule. The recommended dietary allowance for vitamin A is 900 *μ*g and 700 *μ*g per day for adult males and females, respectively [90], with a tolerable upper intake level of 3000 *μ*g per day.

Doses, regimes, methods of extraction, and drug interactions in the setting of a randomized trial must be elucidated to ensure the safety and efficacy of these treatments when given in combination. While we have presented the current evidence on the role of antioxidants on various ocular symptoms, such as dry eyes and presbyopia, one inherent limitation of these studies is the lack of objective outcomes, especially studies investigating presbyopia symptoms.

For example, a decrease in reduced antioxidant levels in the lens may promote the onset of presbyopia [91]. Hesperidin, a major flavonoid in citrus fruits and a natural antioxidant, was found to reduce the number of apoptotic cells, prevent premature cataract symptoms, maintain lens elasticity, and upregulate the mRNA expression of antioxidative enzymes in the selenite-induced cataract rat plasma and lens, hence delaying the formation of nuclear cataract [92] as well as the onset of presbyopia [93]. The challenges of such trials examining antioxidant effects include the influences of diet, environment, and various other patient characteristics, which are difficult to standardize.

Despite having some evidence for specific antioxidants with recommended doses impeding certain age-related eye diseases, there was a lack of extensive studies on the antioxidants' effects on various ophthalmic diseases, and most studies available were concluded within a short period of time. The following table (Table 1) describes the clinical measurements which can be performed to understand the effectiveness of antioxidants on visual function and ophthalmic diseases.

## 3. Conclusion

In conclusion, prevention and treatment of age-related eye diseases with antioxidant substances and other supplements including polyphenol, xanthophyll carotenoids, anthocyanins, and other medicinal plants and natural products [105] have shown promising effects for a multitude of eye diseases. While polyphenols and xanthophyll carotenoids are the most commonly reported natural products used for AMD treatment with positive results, more research and clinical trials are necessary to understand their mechanisms of action.

## Figures and Tables

**Table 1:** Antioxidant effects on vision and measurements to monitor the effects.

Antioxidant effects on	Clinical parameters to be measured	Rationale & method
Refractive error and accommodation	(i) Best-corrected functional near and distant visual acuity	The effect of antioxidants on disease prevention also has an overall impact on vision.
	(ii) The amplitude of accommodation and accommodative status	Bilberry extract influences the progression of myopia and changes in accommodation [83, 85].
Visual fatigue	(i) Subjective measures: visual fatigue questionnaires (ii) Objective measures of indices of visual fatigue: Accommodation parameters, critical flicker–fusion frequency (CFF), and blinking characteristics (iii) Workplace ergonomics questionnaire	Bilberry extract may reduce visual fatigue symptoms [82, 83]. Questionnaires are frequently used to identify self-reported visual fatigue symptoms [94–97]. Workplace ergonomics should take into consideration visual fatigue assessment, as it is one of the biggest confounding factors.
Ocular surface and dry eye	Diagnostic tests to assess and monitor dry eye according to the Tear Film and Ocular Surface Society (TFOS) Dry Eye Workshop (DEWS) II report [92]: (i) Subjective measurement of symptoms: *questionnaires* such as the Ocular Surface Disease Index (OSDI)	Reduction in dry eye symptoms has been reported with intake of oral antioxidants containing bilberry extract [84]. Questionnaire instruments are often self-administered by the patient or research subject without input from the clinician or researcher [98–102].
	(ii) *Tear film stability* (a) Tear film breakup time (TBUT) (b) Noninvasive TBUT (c) Fluorescein tear breakup time (FBUT)	TBUT measures tear film stability. It is defined as the interval of time that elapses between a complete blink and the appearance of the first break in the tear film. The noninvasive technique (NIBUT) is preferred over the fluorescein technique (FBUT) [103].
	(iii) *Tear volume* (a) Schirmer test (b) Phenol red thread (PRT) test (c) Quantitative tear meniscus height and volume	Tear volume is quantified with the Schirmer test. The Schirmer test without anesthesia is well-standardized, providing an estimation of stimulated reflex tear flow. It is performed by folding a Schirmer paper strip at the notch and hooking the folded end over the temporal one-third of the lower lid margin. The score is the measured length of wetting from the notch, after five minutes. It can be performed with (a measurement of basal tear volume) and without topical anesthesia (a measurement of basal plus reflex tear volume) Alternatives to the Schirmer test are the phenol red thread (PRT) test. This test provides an indirect but realistic measure of the resting tear volume. The test is performed by hooking the folded end of the thread in the lower fornix for 15 seconds. When the phenol red meets the alkaline tears, it changes color from white to yellow-orange, yellow, and finally red. The thread is removed after 15 seconds and the red portion will be measured from the very tip regardless of the fold. The quantitative assessment of the tear menisci is, at present, the most direct approach to studying the tear film volume. The tear menisci serve as reservoirs, supplying tears to the precorneal tear film. Tear meniscus may take the form of a height or a cross-sectional volume metric.
	(iv) Damage to the ocular surface (a) Punctate staining of the ocular surface with topical sodium fluorescein	Punctate staining of the ocular surface is a feature of many ocular diseases and instilled dyes are used extensively in the diagnosis and management of dry eye. Ocular surface staining can be assessed using a slit-lamp biomicroscope following instillation of fluorescein dye and viewed under a cobalt blue filter.
	(v) Meibomian gland assessment (a) Meibum quantity, quality, and expressibility	Meibomian glands secrete meibum, which contains components of the lipid layer of the tear film. Meibum quantity, quality, and expressibility are thought to reflect meibomian gland function. The superficial location of the meibomian glands in the tarsal plates permits their anatomic features to be quantified by meibography and confocal microscopy.
	(vi) Objective dry eye measurement (a) Oculus Keratograph® 5M corneal topographer (Germany)	The Oculus Keratograph® 5 M corneal topographer is an advanced corneal topographer with a built-in real keratometer and a color camera. This permits optimized external imaging. Unique features of this instrument include examining the meibomian glands, NIBUT, and the tear meniscus height measurement and evaluating the lipid layer (source: https://www.oculus.de/us/ products/topography/keratograph-5m/oculus-keratograph-5m/). Parameters such as tear film stability, tear meniscus height, blink rate, and meibography can be recorded with this instrument. It provides a quick yet reliable dry eye analysis giving a more accurate clinical diagnosis.
Retinal structure, function, and microcirculation	(i) Optical coherence tomography (OCT) of the macula (non-invasive)	The literature indicates that antioxidant supplements with lutein, zeaxanthin, saffron, and beta-carotene have a reasonable probability of slowing the progression of retinal diseases [15–35, 38–45, 47–49, 53, 54, 66, 67, 69, 86]. OCT of the macula can display a cross-section of the macula, making it an invaluable noninvasive tool to objectively screen, diagnose and monitor AMD.
	(ii) OCT angiography (noninvasive)	OCT angiography is a newer imaging modality using the OCT platform. Real-time scanning of blood corpuscle movements in the retinal vessels produces 3-dimensional images of the macula and optic nerve head vasculature. In pathological conditions characterized by damage to blood vessels such as AMD and DR, this imaging technique also carries the potential for diagnosis, screening, and monitoring. While its advantages include no requirement for peripheral venous canulation and invasive dyes, its effectiveness as compared to conventional angiography is still being investigated.
	(iii) Fundus fluorescein angiography (FFA) and indocyanine green angiography (ICGA) (invasive)	The advantages of conventional dye angiography over OCTA include the ability to determine leakage. While the OCTA can show abnormal vasculature progression, the FFA and ICGA are still necessary to determine the presence of leakage and diagnose polyps in polypoidal choroidal vasculopathy, a subset of AMD. However, it must be used with caution among patients with renal impairment, those who are pregnant or have a history of anaphylaxis.
	(iv) Multifocal electroretinogram (mfERG)	mfERG is an objective test for retinal function by measuring retinal conduction. mfERG can be performed according to guidelines from the International Society for Clinical Electrophysiology of Vision (ISCEV) [104]. The recording is performed with full correction for near vision and dilated pupils. The standard measurement for mfERG amplitude and timing is the amplitude measured from the trough of N1 (first negative deflection) to the peak of P1 (positive peak after N1), and the peak time of P1, respectively.
Optic nerve assessment for structure, function, microcirculation, and risk factor for damage.	(i) Tonometry for IOP measurement	The IOP should be measured as the saffron extract has been reported to cause IOP reduction in glaucoma patients [68]. The IOP is influenced by various factors, including the patient's position during measurement, central corneal thickness, corneal diameter and curvature, the rigidity of the cornea, and the cornea's state of hydration. At present, the gold standard of IOP measurement is the Goldmann applanation tonometry (GAT). Several new devices for IOP measurement have been developed, including noncontact tonometry, the Tono-Pen (Reichert, US), the ICare tonometer (Finland), dynamic contour tonometry (Ziemer, Switzerland), TGDc-01 tonometry (Rjazan State Instrument Making, Russia), and the ocular response analyzer (Reichert, US).
	(ii) Structural test: OCT of the optic nerve	OCT of the peripapillary retinal nerve fiber layer (RNFL) is also useful to diagnose glaucomatous optic neuropathy by virtue of the characteristic loss of the RNFL thickness, particularly at the superior and inferior quadrants of the disc. It is also useful to monitor the progression of optic nerve damage in early to moderate glaucoma. It is less useful in advanced glaucoma, being better monitored by automated perimetry at more advanced stages.
	(iii) Automated perimetry, e.g., Humphrey visual field analyzer (Carl Zeiss Meditech, US)	Automated perimetry is an objective test to map out the visual field defect sustained from various types of optic neuropathy, including glaucoma. Apart from eliciting the characteristic visual field defects, it is also used for characterizing glaucoma into mild, moderate, and severe stages. This allows appropriate and customized treatment of the disease.
